# The invasion history, distribution and colour pattern forms of the harlequin ladybird beetle *Harmonia axyridis* (Pall.) (Coleoptera, Coccinellidae) in Slovakia, Central Europe

**DOI:** 10.3897/zookeys.412.6587

**Published:** 2014-05-29

**Authors:** Ľubomír Panigaj, Peter Zach, Alois Honěk, Oldřich Nedvěd, Ján Kulfan, Zdenka Martinková, Diana Selyemová, Sandra Viglášová, Helen E. Roy

**Affiliations:** 1 Pavol Jozef Šafárik University, Šrobárova 2, Košice, Slovak Republic; 2 Institute of Forest Ecology, Slovak Academy of Sciences, Ľ. Štúra 2, 960 53 Zvolen, Slovak Republic; 3 Crop Research Institute, Drnovská 507/73, 161 06 Prague 6 – Ruzyně, Czech Republic; 4 Faculty of Science, University of South Bohemia, Branišovská 31c, 37005 České Budějovice, Czech Republic; 5 Institute of Entomology, Biology Centre AS CR, Branišovská 31a, 37005 České Budějovice, Czech Republic; 6 Institute of Zoology, Dúbravská cesta 9, Bratislava, Slovak Republic; 7 NERC Centre for Ecology and Hydrology, Wallingford, OX10 8BB United Kingdom

**Keywords:** Alien, altitude, colour morphs, spatial occurrence, spread

## Abstract

The harlequin ladybird beetle *Harmonia axyridis* (Coleoptera, Coccinellidae) has invaded and established in Slovakia. Following unintentional introduction in 2008, the spread of the alien coccinellid was very fast. By the end of 2009, it was recorded across the whole country, and by the end of 2012 it was widely distributed and common in various habitats, particularly gardens, orchards and urban areas, where it was most frequent on trees. The rate of eastward spread was approximately 200 km year^-1^, similar to the overall rate of spread in Europe. Between 2008 and 2012, the coccinellid was recorded in a total of 153 localities, in altitudes ranging from 98 to 1,250 m. Most records of this species were made in lowlands, hilly areas and valleys separating mountain ridges. However, it was only rarely documented in areas above 700 m a.s.l. The non-melanic colour form (f. *succinea*) was dominant along a longitudinal transect including eight urban areas across Slovakia, with the frequency of melanic forms (f. *spectabilis* and f. *conspicua* together) between 6.3 and 19.2% and a median equal to 10.5%. The invasion history and distribution of *H. axyridis* in Slovakia are discussed with regard to the time sequence of records, rate of spread, altitudinal distribution, anthropogenic dispersal, effective recording, proportion of melanic forms and other relevant aspects associated with the spread of this successful invader.

## Introduction

The harlequin ladybird beetle *Harmonia axyridis* (Pallas, 1773) (Coleoptera, Coccinellidae) is native to eastern and central Asia occurring in Japan, Korea, China, Mongolia, Russian Far East through Siberia to Kazakhstan (Brown et al. 2008). This coccinellid is considered an invasive alien species in many countries (Brown et al. 2011). It was intentionally introduced into North America several times from 1916 (Gordon 1985) for biological control of aphids and coccids but was first detected reproducing in the wild decades later, in 1988 (Koch et al. 2006). It has also been introduced in Europe, South America and Africa (Brown et al. 2011). Early introductions of *Harmonia axyridis* into Europe, from Siberia to western Ukraine, were not successful (Kuznetsov 1987). The first intentional introduction of *Harmonia axyridis* in western Europe (from China) was in southern France in 1982, followed by temporary acclimatization by 1991 (Coutanceau 2006). However, *Harmonia axyridis* was recorded in the border area between Belgium and the Netherlands around 2002, representing the extensive spread of this species throughout Europe. It is now known that the European populations of *Harmonia axyridis* originated from hybrids with the invasive population unintentionally introduced from eastern North America (Lombaert et al. 2010). By 2006, the coccinellid was recorded in all regions of Belgium and western Germany, and since 2006 it was frequent in central eastern Germany (Brown et al. 2008). Its continuing eastward spread was immediately documented by the records in Poland in 2006 (Przewozny et al. 2007), followed by rapid establishment there (Ceryngier 2008). First records of the coccinellid in Austria, also, were made in 2006 (Rabitsch and Schuh 2006). In the same year it was first recorded in the Czech Republic (Brown et al. 2008, Špryňar 2008), and within three years (by 2009) it was detected in most areas across the Czech Republic (Nedvěd 2009). In 2008, the beetle was recorded in Hungary (Merkl 2008) and Slovakia (Majzlan 2008, Brown et al. 2011, Zach et al. 2012). By 2009, it was reported from Ukraine and Romania (Nekrasova and Tytar 2009, Marko and Pozsgai 2009).

The invasion history and distribution of *Harmonia axyridis* in some European countries has been detailed in Brown et al. (2008, 2011). However, detailed consideration of the information on the spread and spatial distribution of the insect was missing from Slovakia until 2012. The topography of Slovakia, characterized by a diverse relief, with lowlands, hills and mountains changing on small spatial scales, has enabled first insights into how *Harmonia axyridis* is distributed with altitude and, consequently, this enabled increased understanding of this beetle’s ecological plasticity. Indeed, the altitudinal distribution of coccinellids is only rarely studied (Honěk 1989, Selyemova et al. 2007). Observations of *Harmonia axyridis* from different altitudes, as presented in this study, are unique for Central Europe.

Adult *Harmonia axyridis* exhibit a conspicuous colour pattern polymorphism of the dorsal and ventral side (Blehman 2009), the genetic determination of which has been extensively studied (Sloggett and Honek 2012). In Central Europe, Slovakia included, the species is mostly represented by three forms distinguished by the specific colour pattern of elytra which, in the order of decreasing frequency, are: (1) f. *succinea* with yellow to red background colouration and up to 19 black spots, (2) f. *spectabilis* with black background colouration and four red spots, and (3) f. *conspicua* with black background colouration and two red spots. Also, several rare morphs occur at low frequency such as f. *axyridis* with black background colouration and 12 red spots on the elytra or f. *equicolor* (very rare) with the anterior half of elytra red and posterior black (Hosino 1942, Tan 1946). The size of the spots exhibited by f. *succinea* is determined genetically (Hosino 1942, Tan 1946) and influenced by the temperature during larval and pupal development (Michie et al. 2010). In its native area, there is a conspicuous geographic variation in the proportion of the forms which may correlate with climatic factors (Dobzhansky 1924). The proportion of forms also vary seasonally (Osawa and Nishida 1992, Wang et al. 2009) but not in all geographic areas (Kholin 1990). Despite these advances in our understanding of the colour pattern polymorphism of elytra in *Harmonia axyridis*, in Central Europe little is known about spatial variability in the frequency (proportion) of non-melanic and melanic forms. Further information on colour pattern polymorphism in *Harmonia axyridis* is essential for detecting similarity (and consequently relationships) among the populations of the coccinellid originating from geographically distant areas and/or along the routes of the spread of this invasive alien insect.

This study aims to document the spread (longitudinal and altitudinal distribution) of *Harmonia axyridis* in Slovakia by summarizing the known records of the species collected over the period 2008–2012. Additionally, we document the spatial variation in proportion of the non-melanic and melanic forms of *Harmonia axyridis* to improve understanding of this globally successful invader.

## Methods

### Data recording

The occurrence of *Harmonia axyridis* in Slovakia was recorded over the period 2008–2012 by the authors of this study and other contributors from the Czech Republic and Slovakia, including students of the Pavol Jozef Šafárik University in Košice. The original records collated through the study were supplemented by a few published data (Majzlan 2008).

**Figure 1.**
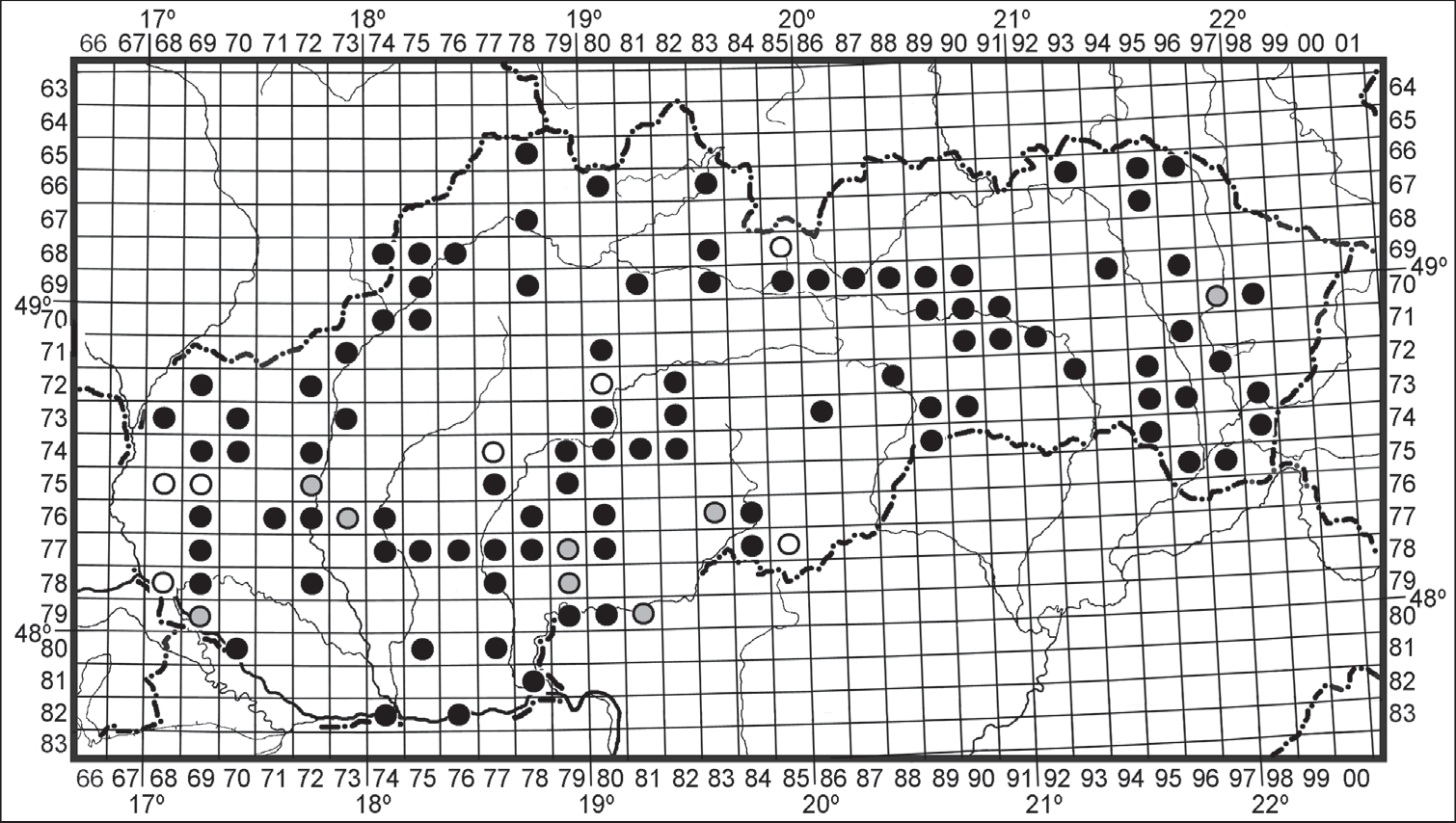
Distribution of *Harmonia axyridis* across Slovakia with the time sequence of the 153 records. Map of the Databank of the Fauna of Slovakia in which each “square” is approximately 11 by 12 km. White circles = 2008, grey circles = 2009, black circles = 2010–2012.

Larvae and adults of *Harmonia axyridis* were recorded by observing them on plants or collecting them from vegetation (various habitats) using a sweep net and/or beating tray. In urban areas, they were also knocked down (by hand) from branches of trees and observed on the bare surfaces of pavements or roads. Species presence was also recorded at overwintering sites, mostly in buildings and, quite uniquely in a man-made stony well, occasionally in sheltered semi-natural habitats such as rock piles covered with dry grass. Sampling effort varied within the study, except for in 2012 when a standardized method (described below) was employed. Most grid squares that have no record of *Harmonia axyridis* (empty in Fig. 1) were not investigated or were sampled randomly with low effort. An exception exists for a few localities for which there were no observations of the species despite intense standardized sampling effort (described below). These negative results are mentioned in the Suppl. material 1.

The non-melanic (f. *succinea*) and melanic forms of *Harmonia axyridis* (f. *conspicua* and f. *spectabilis* together) were recorded along a 380 km long transect extending from the west to the east of the country between 30th July and 7th August 2012, in the following eight urban areas: Bratislava, Nitra, Levice (western Slovakia), Zvolen, Lučenec (central Slovakia), Rožňava, Košice and Michalovce (eastern Slovakia). Along this longitudinal transect adult *Harmonia axyridis* were collected from lower branches of lime trees (*Tilia* spp.) using a circular beating tray (diameter 1.0 m). A total of 30 samples were obtained in each town where one sample represented the individuals from a total of 20 branches of two to five trees; thus the branches in a total length of 600 m were examined for the presence of the coccinellid in each town. Proportions of colour forms, categorized into the same groups as above, were also estimated independently for eastern Slovakia by pooling the observational records from 2010 and 2011.

### Data analysis

The map of distribution of *Harmonia axyridis* in Slovakia was constructed by combining the following data sources: (1) the database of the University of South Bohemia and Institute of Entomology, Biology Centre AS CR (O. Nedvěd et al. 2008–2012), (2) the database of the Pavol Jozef Šafárik University (Ľ. Panigaj et al. 2010–2012) and (3) the database of the Institute of Forest Ecology, Slovak Academy of Sciences (P. Zach et al. 2012). The data (observed presence) were plotted onto the grid map used in the Databank of Fauna of Slovakia (“square” approximately 11 by 12 km). The data from 2008, 2009 and 2010–2012 were plotted separately to demonstrate the temporal sequence of the records.

The distribution of the records of *Harmonia axyridis* by altitude was illustrated by a histogram with breaks (altitude ranges) set to 100 m. Frequencies of non-melanic forms of the coccinellid were compared among particular towns along the longitudinal transect (for each combination of towns possible) using the chi-square test (2×2 contingency table) (Zar 2010). Statistical calculations were made in the program STATISTICA, version 9.0 (Statsoft, Inc. 2008).

## Results

A total of 153 records of *Harmonia axyridis* were made across Slovakia over the period 2008–2012, providing evidence of the occurrence of the coccinellid within 109 squares (11 × 12 km) across Slovakia, i.e. 25.5% of all available squares (Fig. 1, Suppl. material 1).

In 2008, *Harmonia axyridis* was recorded in seven localities in western, central and northern Slovakia. In 2009, it was known from another eight localities, one of them in eastern Slovakia. Between 2010 and 2012, it was reported from numerous other localities across the entire country. The coccinellid was observed in lowland and submontane areas along the south of the country, extending northwards into long valleys of Váh, Hron and Hornád rivers (Fig. 1).

**Figure 2.**
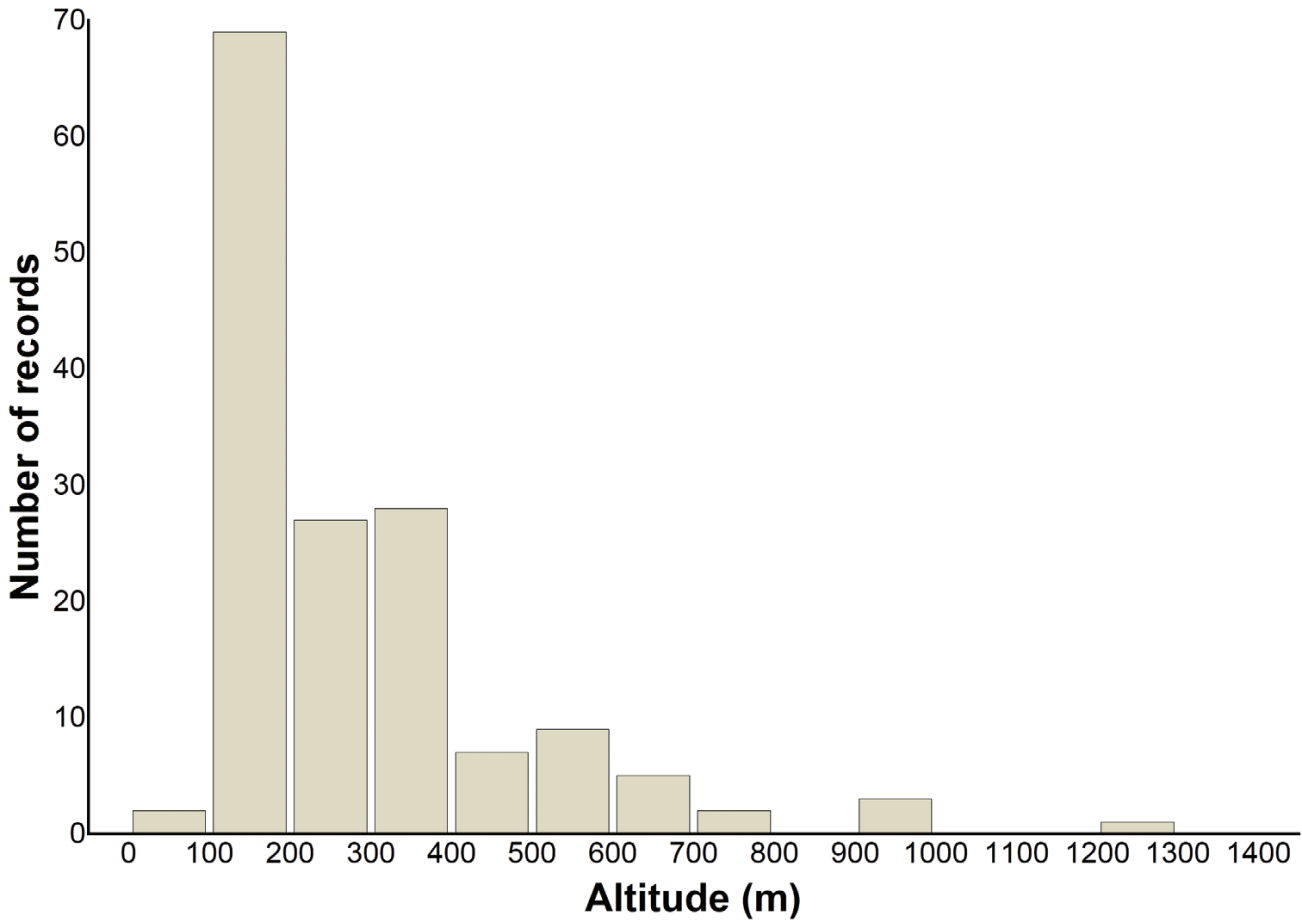
Frequency distribution of the records of *Harmonia axyridis* from Slovakia, 2008–2012 (n = 153). Data arranged by altitude (m).

The majority of records of *Harmonia axyridis* come from lowlands (up to 200 m a.s.l.) and hilly areas (200–700 m a.s.l.). The records from altitudes above 700 m, and especially those from mountain areas above 1,000 m a.s.l., are much less frequent (6 and 1 respectively) than those from the lowlands and hilly areas (Fig. 2).

**Figure 3.**
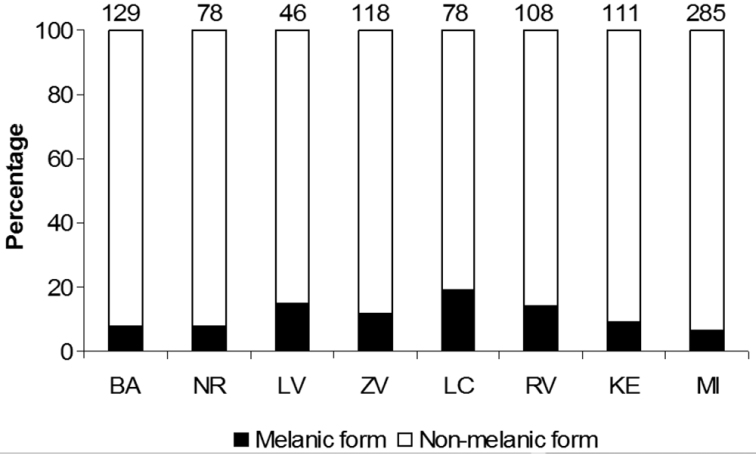
Percentage of melanic f. *spectabilis* and f. *conspicua* (combined) and a non-melanic f. *succinea* among adult *Harmonia axyridis* (n = 953) in eight urban areas along the 380 km long west-east transect across Slovakia. BA – Bratislava, NR – Nitra, LV – Levice, ZV – Zvolen, LC – Lučenec, RV – Rožňava, KE – Košice, MI – Michalovce. 30th July–7th August 2012

Three colour forms (f. *succinea*, f. *spectabilis*, f. *conspicua*) were documented among the 953 adult *Harmonia axyridis* collected in eight urban areas (towns) located along the 380 km long west-east transect, sampled between 30th July–7th August 2012. The proportion of melanics varied from 6.3% to 19.2% (Fig. 3), with a median of 10.5%. The frequency of melanic forms at Lučenec (19.2%) was significantly different from the frequencies at Bratislava (7.8%) (chi-square test, χ^2^=4.999, df=1, p=0.025) and Michalovce (6.3%) (chi-square test, χ^2^=10.846, df=1, p<0.001). Frequencies of melanic forms in the other six towns did not differ significantly from each other (chi-square test, p>0.05). Pooled samples from eastern Slovakia (2010–2011) consisted of a total of 410 specimens, the 59 (14.4%) of which were melanic (f. *spectabilis* only).

## Discussion

*Harmonia axyridis* has recently been described as the fastest-spreading invasive alien insect in Europe (Brown et al. 2011). In Slovakia, it was first recorded in Malaise traps in Tatra Mountains (Tichá dolina valley and Podbanské area) in 2008 (map grid number 6885, see Fig. 1 and Suppl. material 1; collector O. Majzlan), and in the same year records were reported from Banská Bystrica (7280, V. Franc), Malacky (7568, R. Hergovits), Bratislava (7868, R. Hergovits), Župkov (7477, K. Bucsek) (Majzlan 2008), Sološnica (7569, T. Stacho) and Šikov (7785, O. Pultar). The records reported in this study show that as early as two years after introduction (by the end of 2009) the coccinellid was distributed across the entire country and that it remained abundant through 2012. The time sequence of the records indicates easterly spread of the coccinellid, with introductions most likely from the Czech Republic and/or Austria (Brown et al. 2011, Nedvěd 2009; Rabitsch and Schuh 2006), although the spread from the other neighbouring countries, Hungary and Poland, cannot be excluded. Observations of *Harmonia axyridis* became more frequent between 2010 and 2012 as its distribution got wider and recording effort increased. We are aware that scarcity of records between 2008 and 2009 (Fig. 1) may be due to lower recording effort rather than real absence of the coccinellid in the country at that time. However, we did not observe *Harmonia axyridis*, despite intensive surveys at several localities, in central and eastern Slovakia between 2008 and 2010, but it was subsequently recorded in 2012.

Undoubtedly, *Harmonia axyridis* has spread very quickly across Slovakia since its arrival in 2008 (Brown et al. 2011). Similar rapid spread of the insect was observed in the Czech Republic (Nedvěd 2009), Poland (Ceryngier 2008; with late arrival and relative scarcity in north-eastern Poland (http://www.cbe-pan.pl/harmonia/rozmieszczenie.htm) and Hungary (Markó and Pozsgai 2009). However, we did not observe this species in Estonia in the summer of 2012 (O. Nedvěd, unpublished data). The cold climate there may prevent successful establishment of *Harmonia axyridis*, but it also does not appear to occur in hot climates, except for one record in Kenya (Nedvěd et al. 2011).

*Harmonia axyridis* is considered a successful invader (Koch et al. 2006, Brown et al. 2011) and is known to spread at rates between 100 and 500 km year^-1^, depending on area and/or whether its introduction was assisted by anthropogenic means (Brown et al. 2011). According to McCorquodale (1998) the rate of *Harmonia axyridis* range expansion in eastern North America was as fast as 442 km year^-1^, reflecting both the innate dispersal capability and human mediated movement of the coccinellid (Koch et al. 2006). Brown et al. (2011), taking Europe as a whole, have calculated the maximum rate of the spread of *Harmonia axyridis* to be approximately 200 km year^-1^. Taking this rate and the eastward direction of spread into consideration, the insect was predicted to colonize much of Slovakia (distance between western and easternmost part of the country is approximately 400 km) within two years. The distribution and time sequence of the records reported here (Fig. 1), together with many negative observations in the eastern part before 2010, support this prediction.

The spread of *Harmonia axyridis* may be assisted and, indeed, accelerated by human movement. For example, an adult specimen of the beetle was unintentionally transported in a coach from Prague (western Czech Republic) to Žarnovica (central Slovakia), travelling over the direct distance of 355 km in 7 hours (P. Zach, personal observation). Human-assisted long-distance dispersal, deliberate and inadvertent, has been well-documented in the case of *Harmonia axyridis* (Harmon et al. 2007) and such transportation has clearly played a dominant role in the fast spread of this coccinellid throughout the world (Lombaert et al. 2011). The strong preference of *Harmonia axyridis* for urban environments (the species is frequently found in buildings and/or on ornamental trees in built up areas) increases the probability of being dispersed by humans. The outlying (spatially separated) record of the coccinellid in Ukraine (Kyiv) from 2009 (Nekrasova and Tytar 2009) suggests an anthropogenic origin. Passive transportation by anthropogenic means is the mechanism resulting in rapid spread of the beetle (Koch et al. 2006). *Harmonia axyridis* is known to exhibit high fecundity and long lasting fertility and, therefore, a single fertilized female may establish a new large colony without additional access to a male (Awad et al. 2013).

The success in recording *Harmonia axyridis* strongly depends on the establishment and abundance of the beetle in a certain habitat or area. In Slovakia, this semi-arboreal coccinellid (Hodek and Honek 1996) was reported as established immediately after the introduction in 2008 (O. Nedvěd and M. Marko in Brown et al. 2011). Based on the data from the U.S.A. and England, *Harmonia axyridis* may become a dominant species of coccinellid communities within four to five years following introduction (Majerus et al. 2006). In 2012, five years after the introduction of *Harmonia axyridis* in Slovakia, the relative abundance of the beetle in coccinellid communities of lime trees in eight towns along the longitidinal transect of southern Slovakia was in the range from 47.9 (Levice) to 92.2% (Michalovce) with the median 71.7% (P. Zach, A. Honek, J. Kulfan, Z. Martinková, D. Selyemová, unpublished data, 30th July–7th August 2012). In central Europe, a combination of lime trees and urban areas enables very efficient recording of *Harmonia axyridis*. In late summer 2012, the beetle was found in all the urban areas surveyed and always on lime trees. Even a solitary lime tree present at a rural site enabled collecting of *Harmonia axyridis* with minimal effort (by knocking one to five branches only) (e.g. in Tatranská Štrba, Z. Martinková, A. Honěk, D. Selyemová, J. Kulfan, P. Zach, personal observation).

In Slovakia, as well as in the Czech Republic (Honek et al. 2013), *Harmonia axyridis* was documented in various habitats and on many plant species (Zach et al. 2013). This habitat generalist (Brown et al. 2011) was often recorded in urban areas with deciduous trees and in gardens or orchards with fruit trees. These habitats harbour essential (aphids and coccids) and alternative food (fruits) of the beetle (Hodek and Evans 2012). The local topography, also, is known to play a crucial role in the spread and distribution of the coccinellid (Honek 2012). The records reported here indicate spatial heterogeneity with most records from within the catchment area of the central flow of the river Váh in the west or within the catchment area of the river Hornád in the east of the country. There are also scattered records from non-urban habitats along the middle flow of the river Hron (Fig. 1). Valleys of greater rivers in Slovakia are densely populated locally, with abundant road and rail traffic. These open corridors in fragmented landscapes are crucial for the spread and establishment of *Harmonia axyridis*.

During field surveys, *Harmonia axyridis* was recorded in altitudes ranging from 98 m in the south-east to 1,000 m in the north (foothills of High Tatra Mountains), or as high as 1,250 m in the central part of the country (Poľana Mountains). Most often, however, it was reported from lowlands, hilly areas and valleys separating mountain ridges, in altitudes bellow 700 m. The low number of records of the coccinellid below 100 m is a consequence of the relief – the lowest point in Slovakia being 94.3 m a.s.l. In contrast, few records were obtained from altitudes above 700 m. This might be a consequence of the possible scarcity of the beetle in higher altitudes and/or difficulty to detect it, especially in inaccessible and not frequently surveyed mountain areas – the highest point in Slovakia being 2,654 m. It is unclear at the moment as to whether fragmented habitats in mountain ranges dominated by Norway spruce [*Picea abies* (L.) Karst.] play any role in the active spread of the coccinellid, as documented for native species of coccinellids (Selyemová et al. 2007). The uppermost record of five individuals of *Harmonia axyridis* in a trailer (Poľana Mountains, 1,250 m) requires special consideration as it might result from anthropogenic dispersal. The regions with occurrence of the beetle are determined by the density of occupied neighbouring squares. The differences in sampling effort is thus averaged and compensated. There are other examples of studies on the distribution of *Harmonia axyridis* based on grid mapping using “citizen science“ approaches with unknown sampling effort (Roy et al. 2012).

Coccinellids in Central Europe overwinter as adults, often in large aggregations (Honěk 1989), which is also the case for *Harmonia axyridis* (Zach et al. 2013). The aggregations of overwintering specimens of *Harmonia axyridis* were commonly found in anthropogenic habitats in western, central and eastern Slovakia, mostly in buildings (clusters of 2 to 42 individuals, n = 12) or outer splits in building walls (5 and 7 individuals) (P. Zach personal observation) and a human-made stony well (more than 846 individuals) (Svidník, D. Jurina, personal observation). Very occasionally records were obtained from sheltered semi-natural habitats like rock piles covered with dry grass (7, 15 and 18 aggregated individuals). Since *Harmonia axyridis* is considered as a nuisance species to humans, specifically in built up areas (Koch and Galvan 2008), the clusters of aggregated specimens in buildings, often, are unstable due to removal by man (e.g. at Sliač spa – frequent complains of spa visitors in October 2011 and 2012; P. Zach, unpublished data). European populations of *Harmonia axyridis* have weak diapause (Raak-van den Berg et al. 2012). Individuals leave aggregations in warm (heated) buildings between January and March, starve and die because of the lack of food (M. Mikuš, O. Nedvěd, P. Zach, personal observations). Aggregations in semi-natural habitats with cool microclimates seem more stable than those associated with heated buildings with regard to artificial removal and mortality (Raak-van den Berg et al. 2012; O. Nedvěd, P. Zach, personal observations). Aggregating behaviour plays an important role in the anthropogenic spread of *Harmonia axyridis* (Labrie et al. 2008). Indeed, 200 individuals of the species in Åndalsnes, Norway, in 2007, were found on timber imported from North America (Lombaert et al. 2010). Nevertheless, no evidence of human-assisted transportations of aggregated individuals of the coccinellid has been obtained during our surveys.

The proportion (percentage) of melanic and non-melanic individuals in urban populations of *Harmonia axyridis*, sampled along the longitudinal transect over a short time period in the summer of 2012, were reasonably consistent, although some spatial variability was evident: the range was 6.3–19.2% and median was 10.5%. The estimate of the percentage of melanic individuals made for eastern Slovakia (14.4%) was within this range. The proportion of melanic forms detected along this transect was somewhat lower than that in Belgium, England and Luxembourg but it was similar to those observed in the Czech Republic (Brown et al. 2008, Nedvědová et al. 2013). Nevertheless, deviations from the rather consistent pattern of colour form frequency across Europe (Brown et al. 2008), detected in the study (Fig. 3), are not excessive. This perhaps indicates (in a broader sense) genetic similarity of individuals spreading from few points of origin in western Europe but genetical analysis is needed (Zakharov et al. 2011) to clarify this in detail. Proportions of the two melanic forms were not recorded separately during the survey. In Slovakia, f. *spectabilis* is more frequent than f. *conspicua*, which is also the case in the Czech Republic (Nedvědová et al. 2013).

## Conclusions

The detailed observations documented here provide further evidence of invasion by *Harmonia axyridis* within Europe. The ability of *Harmonia axyridis* to exploit a range of altitudes provides further insights into the spatial spread of this species. Indeed, the spectacular spread of *Harmonia axyridis* around the world is testament to the success of this invader. The threat to biodiversity posed by this generalist beetle has been widely recognised (Roy et al. 2012). Additionally characteristics of this species which have ensured its success as an invader have been given consideration (Roy and Wajnberg 2008; Soares et al. 2008; Comont et al. 2012). The opportunity to increase our understanding of the ecology of *Harmonia axyridis* is critical for ensuring lessons are learnt from this invasion process.

## Supplementary material

Supplementary material 1 Records of *Harmonia axyridis* in Slovakia doi: 10.3897/zookeys.412.6587.app1 Data type: occurence Explanation note: There are records from 2008 to 2012; years 2008 and 2009 marked in bold. The localities (sites of collection) are arranged in alphabetical order. Indicated are the numeric code of the mapping square (see Fig. 1), year of the first record and name of observer. Data on absence of the species are indicated by subsequent records of the species in the same squares or near squares from which it had not previously been recorded (e.g. 2008 – no record, 2009 – first record). This dataset is made available under the Open Database License (http://opendatacommons.org/licenses/odbl/1.0/). The Open Database License (ODbL) is a license agreement intended to allow users to freely share, modify, and use this Dataset while maintaining this same freedom for others, provided that the original source and author(s) are credited.Ľubomír Panigaj, Peter Zach, Alois Honěk, Oldřich Nedvěd, Ján Kulfan, Zdenka Martinková, Diana Selyemová, Sandra Viglášová, Helen Roy
