# Supplementary material for: The invasion history, distribution and colour pattern forms of the harlequin ladybird beetle Harmonia axyridis (Pall.) (Coleoptera, Coccinellidae) in Slovakia, Central Europe
Source: Zookeys. 2014 May 29;(412):89–112. doi: 10.3897/zookeys.412.6587 (PMC4042697; doi:10.3897/zookeys.412.6587)
Supplement: Supplementary material 1 — Records of Harmonia axyridis in Slovakia [file zookeys-412-089-s001.doc]

**Records of *Harmonia axyridis* in Slovakia**

There are records from 2008 to 2012; years 2008 and 2009 marked in bold. The localities (sites of collection) are arranged in alphabetical order. Indicated are the numeric code of the mapping square (see Fig. 1), year of the first record and name of observer. Data on absence of the species are indicated by subsequent records of the species in the same squares or near squares from which it had not previously been recorded (e.g. 2008 – no record, 2009 – first record).

Bajč (8075/2010, O. Pultar), Bajtava (8178/2010, O. Pultar), Banská Bystrica (7280/**2008**, V. Franc; 2010, O. Nedvěd), Banská Bystrica – Jakub (7280/2012, P. Zach), Banská Bystrica – Kordíky (7280/2012, A. Krištín), Banská Štiavnica (7579/2012, A. Krištín), Bardejov (6693/2011, T. Jászay), Bátovce (7778/2012, P. Zach), Belá nad Cirochou (7098/2011, Ľ. Panigaj et al.), Beluša (6975/2011, Ľ. Panigaj et al.), Bodíky (8070/2010, Ľ. Panigaj et al.), Bolešov (7074/2011, Ľ. Panigaj et al.), Bratislava (7868/**2008** K. Hergovits; 2012, P. Zach), Brusno (7282/2011, Ľ. Panigaj et al.), Bučany (7572/2011, O. Majzlan), Cerov (6876/2011, Ľ. Panigaj et al.), Čabradský Vrbovok (7780/2011, Ľ. Panigaj et al.), Čadca (6578/2010, O. Nedvěd), Čachtice (7272/2010, Ľ. Panigaj et al.), Čata (8077/2012, P. Zach), Čičarovce (7498/2011, Ľ. Panigaj et al.), Čifáre (7776/2012, P. Zach), Detva-Zapriechody (7482/2012, M. Mikuš), Devínska Kobyla-Sandberg (7868/2012, A. Krištín), Dolná Seč (7877/2012, P. Zach), Domaša (6996/2012, Ľ. Panigaj et al.), Dubnica n/V. (7075/2011, Ľ. Panigaj et al.), Dudince (7879/**2009**, O. Pultar), Dunajská Lužná (7969/**2009**, O. Pultar), Dvory nad Žitavou (8075/2010, O. Pultar), Fiľakovo (7784/2011, 2012, P. Zach), Galanta (7872/2012, D. Selyemová), Gelnica (7191/2011, Ľ. Panigaj et al.), Hlohovec (7572/**2009**, M. Klekner et I. Navrátilová), Humenné (7097/**2009**, K. Darnadyová), Chmeľovec (6994/2012, Ľ. Panigaj et al.), Ipeľské Predmostie (7980/2012, P. Zach), Ivánka pri Dunaji (7869/2010, Ľ. Panigaj et al.), Jabloňovce (7678/2012, P. Zach), Jaklovce (7191/2012, Ľ. Panigaj et al.), Jalovec (6883/2011, V. Hemala), Janíkovce pri Nitre (7774/2012, P. Zach), Júr nad Hronom (7877/2012, P. Zach), Kalša (7395/2011, A. Mock), Kazimír (7495/2010, Ľ. Panigaj), Kišovce (6988/2012, Ľ. Panigaj et al.), Kľačany (7673/**2009**, O. Pultar), Komárno (8274/2012, P. Zach), Kostolná-Záriečie (7173/2011, Ľ. Panigaj et al.), Košice-Čermeľ (7293/2011, Ľ. Panigaj), Košice (7293/2010, Ľ. Panigaj), Kováčová (7480/2012, P. Zach), Kováčovo (8178/2012, P. Zach), Kožuchovce (6696/2011, Ľ. Panigaj et al.), Kráľovský Chlmec (7597/2012, Ľ. Panigaj et al.), Kravany nad Dunajom (8276/2010, 2012, A. Hoňek), Krompachy (7091/2011, Ľ. Panigaj et al.), Krupina (7680/2010, O. Nedvěd), Kúty (7368/2012, P. Zach), Ladmovce (7596)/2011, Ľ. Panigaj), Lakšárska Nová Ves (7469/2012, M. Holecová), Levice (7777/2012, P. Zach), Levoča (6989/2012, Ľ. Panigaj et al.), Liptovský Mikuláš (6983/2011, Ľ. Panigaj et al.), Lučenec (7684/2012, P. Zach), Lukové pri Zvolene (7480/2012, P. Zach), Malacky (7568/**2008**, K. Hergovits), Martin (6978/2011, Ľ. Panigaj et al.), Michalovce (7297/2012, P. Zach, J. Kulfan, M. Veľký, M. Mikuš), Moča (8276/2012, P. Zach), Modra – Vinosady (7669/2012, M. Holecová), Mojmírovce (7774/2010, Ľ. Panigaj et al.), Moravany nad Váhom (7373/2011, J. Petrovský), Nebrová - Kysuce (6874/2012, A. Krištín), Nitra (7674/2011, Ľ. Panigaj et al.), Nitra – Zobor (7674/2012, A. Krištín), Nižná Slaná (7288/2012, Ľ. Panigaj), Nová Baňa (7577/2012, P. Zach), Nové Mesto nad Váhom (7272/2012, P. Zach, J. Kulfan, M. Parák), Očová (7481/2012, J. Váľka), Opatovská Nová Ves (7683/**2009**, M. Klekner et I. Navrátilová), Ostratice (7375/2011, O. Pultar), Pezinok (7769/2011, Ľ. Panigaj et al.), Piešťany (7472/2011, O. Pultar), Plášťovce (7879/2012, A.Krištín), Podbanské-Tichá dolina (6885/**2008**, O. Majzlan), Podbanské (6885/2012, J. Kulfan, P. Zach), Poľana – chata ZŤS (7382/2012, A. Krištín), Poprad (6987/2012, Ľ. Panigaj et al.), Poráč (7190/2012, P. Zach), Poša (7196/2012, Ľ. Panigaj et al.), Púchov (6875/2011, Ľ. Panigaj et al.), Revúca (7386/2010, O. Nedvěd), Rimavská Sobota (7686/2011, Ľ. Panigaj et al.), Rohožník (7569/2010, Ľ. Panigaj et al.), Rožňava (7389/2012, P. Zach, J. Kulfan, M. Veľký), Rudňany (7190/2012, P. Zach), Ružín (7192/2011, Ľ. Panigaj), Ružomberok (6981/2010, O. Nedvěd), Sečovce (7295/2010, 2012, J. Petrovský), Sekule (7368/2011, Ľ. Panigaj et al.), Selešťany (7981/**2009**, M. Klekner et I. Navrátilová), Senica (7370/2012, P. Zach), Senné (7398/2012, A. Krištín), Silica (7489/2010, T. Doležal), Sirk (7386/2011, Ľ. Panigaj), Sliač – kúpele (7380/2012, P. Zach), Smižany (7089/2012, B. Endel), Smolenice (7470/2012, P. Zach), Smolinské (7368/2012, P. Zach), Smrdáky (7269/2012, P. Zach), Snina (7098/2012, Ľ. Panigaj et al.), Sološnica (7569/**2008**, T. Stacho), Spišská Nová Ves (7089/2009 – no record, O. Nedvěd; 2012, P. Zach), Spišské Podhradie - Spišský hrad (6990/2012, P. Zach), Spišské Vlachy (7090/2011, T. Jászay), Starý Hrádok (7877/2012, P. Zach), Stropkov (6795/2011, Ľ. Panigaj), Súdovce (7779/**2009**, M. Klekner et I. Navrátilová), Svätá Mária (7597/2011, A. Mock), Svätojurský Šúr (7769/2010, O. Majzlan ), Svidník (6695/2011, Ľ. Panigaj; 2012, D. Jurina), Šahy (7979/2010, O. Nedvěd), Šamorín (7969/2010, Ľ. Panigaj et al.), Šarišská Poruba (6994/2011, Ľ. Panigaj), Šaštínske Stráže (7368/2012, P. Zach), Šikov (7785/**2008**, O. Pultar), Špania dolina (7180/2012, P. Zach), Štúrovo (8178/2012, P. Zach), Tatranská Štrba (6986/2012, Z. Martinková, A. Honěk, D. Selyemová, J. Kulfan, P. Zach), Tekovské Lužany (7877/2012, P. Zach), Tisinec (6795/2011, Ľ. Panigaj et al.), Trebišov (7396/2011, Ľ. Panigaj), Trnava (7671/2012, P. Zach), Tvrdošín (6683/2011, Ľ. Panigaj et al.), Veľké Kapušany (7498/2011, Ľ. Panigaj), Veľké Kozmálovce (7777/2012, P. Zach), Vinohrady nad Váhom (7672/2012, D. Selyemová), Vinosady – Holubyho (Pezinská) lesostep (7669/2012, M. Holecová), Vráble (7775/2012, P. Zach), Východná – Tatry (6985/2012, M. Koreň), Vychylovka (6680/2012, M. Parák), Vyškovce nad Ipľom (7979/2012, P. Zach), Zádiel (7390/2010, T. Doležal, O. Nedvěd), Zatín – Zatínsky luh (7597/2012, M. Holecová), Zvolen (7480/2010, O. Nedvěd, J. Kulfan), Zvolenská Slatina (7481/2012, P. Zach), Žakarovce (7191/2012, Ľ. Panigaj et al.), Žemliare (7877/2012, P. Zach), Žiar nad Hronom (7479/2012, P. Zach), Žilina (6778/2011, Ľ. Panigaj et al.), Žilina – Závodie (6778/2012, P. Zach), Župkov (7477/**2008**, K. Bucsek).
